# The Future Life

**Published:** 1889-02

**Authors:** Victor Hugo


### THE FUTURE LIFE.

I feel in myself the future life. I am like a forest which has been more than once cut down. The new shoots are stronger and livelier than ever. I am rising, I know, toward the sky. The earth gives me a generous sap, but heaven lights me with the reflection of unknown worlds.

You say the soul is nothing but the resultant of bodily powers , why then is my soul the more luminous when my bodily powers begin to fail ? Winter is on my head and eternal spring is in my heart. Then I breathe, at this hour, the fragrance of the lilies, the violets, and the roses as at twenty years.

The nearer I approach the end, the plainer I hear around me the immortal symphonies of the worlds which unite me.

It is marvelous, yet simple. It is a fairy tale, and it is a history. For half a century I have been writing my thoughts in prose, verse, history, philosophy, drama, romance, tradition, satire, ode, song—I have tried all. But I feel that I have not said the thousandth part of what is in me. When I go down to the grave I can say, like so many others, "I have finished my day's work ;" but I cannot say, "I have finished my life." My day's work will begin again the next morning. The tomb is not a blind alley, it is a thoroughfare. It closes in the twilight to open with the dawn.

I improve every hour because I love this world as my fatherland. My work is only a beginning. My work is hardly above a foundation. I would be glad to see it mounting and mounting forever. The thirst for the infinite proves infinity.—*Victor Hugo.*

